# Rectification of Bowl-Shape Deformation of Tidal Flat DEM derived from UAV Imaging

**DOI:** 10.3390/s20061602

**Published:** 2020-03-13

**Authors:** Hyoseong Lee, Dongyeob Han

**Affiliations:** 1 Department of Civil Engineering, Sunchon National University, 255 Jungangro, Suncheon, Jellanamdo 57922, Korea; hslee@scnu.ac.kr; 2 Department of Civil Engineering, Chonnam National University, 77 Yongbongro, Bukgu, Gwangju 61186, Korea

**Keywords:** tidal flat, ground control points, a least-squares height-difference DEM matching, polynomial model, bowl effect

## Abstract

It is necessary to periodically obtain topographic maps of the geographical and environmental characteristics of tidal flats to systemically manage and monitor them. Accurate digital elevation models (DEMs) of the tidal flats are produced while using ground control points (GCPs); however, it is both complicated and difficult to conduct GPS surveys and readings of image coordinates that correspond to these because tidal flat areas are not easy to access. The position and distribution of GCPs affect DEMs, because the entire working area cannot be covered during a survey. In this study, a least-squares height-difference (LHD) DEM matching method with a polynomial model is proposed to increase the number of DEM grids while using a presecured precise DEM to rectify the distortion and bowl effect produced by unmanned aerial vehicle (UAV) images. The most appropriate result was obtained when the translation parameters were quadratic curve polynomials with an increasing number of grids and the rotation parameters were constant. The experimental results indicated that the proposed method reduced the distortion and eliminated the error caused by the bowl effect while only using a reference DEM.

## 1. Introduction

Coastal areas are vulnerable regions that are easily affected by natural and artificial environmental changes. There is growing interest in these areas in a number of associated fields, as various forms of land (sediment) have either emerged or disappeared, owing to these changes [1,2]; a unique ecological environment among these is the intertidal flat. The intertidal flat is submerged in water during high tide and its surface is fully exposed during low tide. It also possesses significant features in terms of habitat for shellfish, water purification, maintenance of diverse species, flood control, and recreational and scenic resources [3]. The Korean intertidal flat is one of the largest tidal flats in the world. It is also an internationally protected shelter for endangered migratory birds and is considered to be one of the prosperous ecosystems inhabited by various species living in clusters [4].

It is necessary to periodically obtain topographic maps that understand the geographical and environmental characteristics of tidal flats to systemically manage and monitor them. The bottom topography of an intertidal flat in a time series manner can be used, particularly to estimate the annual or seasonal variations in the sediment budget and coastal changes [5,6,7]. Very few investigations have been carried out so far despite the importance of intertidal flats, as access to such areas is restricted. An intertidal flat consists of inflow of seawater, soil with no solidification, and deposits of mud and silt. Therefore, non-intrusive and temporally regular measurements over intertidal flats are essential.

Until now, the topography of tidal flats was obtained by using either airborne light detection and ranging (LiDAR) or echo sounders [8,9]. In 1999, Irish et al. introduced a scanning hydrographic operational airborne lidar survey (SHOALS) that could simultaneously measure the depth of water and terrain of the coastal area [10]. Measurements using the airborne LiDAR system incurred high costs and involved a number of processing tasks. Moreover, the system did not work adequately in the case of tidal flats, as they contain remnants of water and puddles of sea water [5,11]. The use of echo sounders was also not suitable, as the ship carrying the hull-mounted sonar could not conduct the survey in conditions where the water-level was very shallow, such as in intertidal flats. Owing to these limitations, the waterline method was widely utilized to generate the topography of tidal flats [12,13]. It is known that the waterlines extracted from optical and SAR satellite images can be stacked to construct contour lines corresponding to the height of the tide at the time of passage of the satellite [6]. However, this method requires a large amount of satellite data that are acquired in a short period of time and at different tidal heights, as well as very accurate records of tidal gauge data. Moreover, in 2007, Kim et al. reported that the waterlines extracted from SAR images may be different from the actual waterlines, depending on the radar frequency used [14]. Consequently, the above-mentioned methods were not suitable for generating the bottom topography of intertidal flats.

In recent times, a number of studies were conducted to assess the possibility of generation of the topography of tidal flats using unmanned aerial vehicle (UAV) images and photogrammetric software, such as Agisoft PhotoScan or Pix4D. According to these studies, on-site GCPs were required for the accurate development of digital elevation model (DEM) [2,7,15]. However, a survey of the GCPs was difficult, as it was not easy to approach the tidal flats in the Southwest coast of the Korean peninsula, where the proportion of silt and clay content were high (http://www.ecosea.go.kr) [16].

Without GCPs, the DEM generated from the UAV images and photogrammetric software probably have a nonlinear distortion that overestimated the elevation of the terrain. The systematic overvaluation of the terrain increased as the distance from the center increased, due to the ‘bowl or doming effect’ [17,18], which affected the external part of the area that is covered by the GCPs [19]. Recently, UAVs with an integrated on-board real-time kinematics (RTK) have been used for mapping terrain. In an area of about 550 × 330 m^2^ containing buildings, roads, and meadows, the average DSM accuracy had a ground sampling distance that was obtained while using SenseFly eBee-RTK was approximately 3.7 without the GCPs [20]. However, an assessment of the accuracy of the DEM shows an average root mean square error (RMSE) of approximately 2 m without GCPs when a DJI phantom 4 RTK is flown on a north-to-south trajectory over a 2 km coastal site [21]. The base station must be placed even if no GCP has to be surveyed, especially in case of inaccessible areas.

This study proposes an least-squares height-difference (LHD) DEM matching technique with a polynomial model to correct the distortion in the DEM produced by the UAV, while using the existing reference DEM such as LiDAR or pre-compensated dense DEM instead of the GCPs. The LHD technique is based on the height difference in the same-plane positions between the two DEMs for the calculation of seven three-dimensional transformation parameters (translations, rotation angles, and scale) [22,23,24]. However, the distortion due to the bowl effect could not be removed, even if the DEM produced by the UAV images can use the reference DEM to correct the these parameters. Therefore, an LHD matching technique with a polynomial model was developed for refinement of the distorted DEM. This approach determines the polynomial coefficients that depend on the DEM grid lines for transformation parameters.

The remainder of this paper is organized, as follows: The fundamentals of the proposed LHD matching are introduced in Section 2, with an explanation of the various input parameters. Section 3 details the fields of experiment, data processing, and instrumentation. Section 3 also discusses the experimental results, where the analysis focuses on the enhancement of accuracy of the corrected DEM. Finally, Section 4 presents the conclusions.

## 2. Proposed Approach of LHD Matching

A UAV DEM built without GCPs usually contains a deformation, owing to the bowl effect. The bowl effect is probably affected by location errors in the tie-point that is used by the software for self-calibration and aerial triangulation. Therefore, a non-metric camera with inaccurate interior orientation parameters (IOPs, i.e., focal length, principal point displacement, and lens distortion) is mounted on the UAV and the exterior orientation parameters (EOPs, i.e., position and rotation of the principal point) are determined by errors that are propagated by the bundle block adjustment process between overlapping images when a camera model with initially inaccurate values is used [17,18,19].

**Figure 1:**
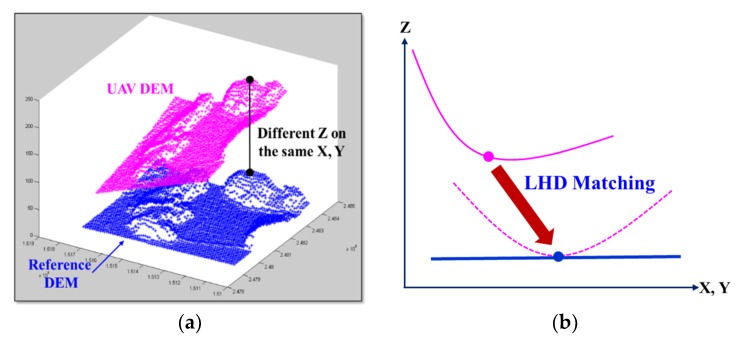
Least-squares height-difference (LHD) matching between a reference digital elevation model (DEM) and a unmanned aerial vehicle (UAV) DEM from an UAV (**a**) and a common result from the UAV DEM, as affected by the bowl effect (**b**).

The purpose of our study was to rectify the DEM that is produced by the overlapping UAV images and photogrammetric software using LHD matching, because it is difficult to collect GCPs via field surveys in an inaccessible area, such as a tidal flat. We used only the ground coordinates of the pre-established reference DEM, in particular, to apply the LHD. The heights of the DEM were extracted by determining same-plane positions within a grid interval in both the reference and produced DEMs, as shown in Figure 1.

Without the GCPs, a bowl-shaped deformation cannot be rectified, even by applying the LHD matching method while using the seven transform parameters and reference DEM (Figure 1). We proposed a new LHD matching technique that was applied by using polynomial models of the transformation parameters as functions of the number of grid spaces between the two DEMs to overcome this problem (Equation (1)).

**Figure 2:**
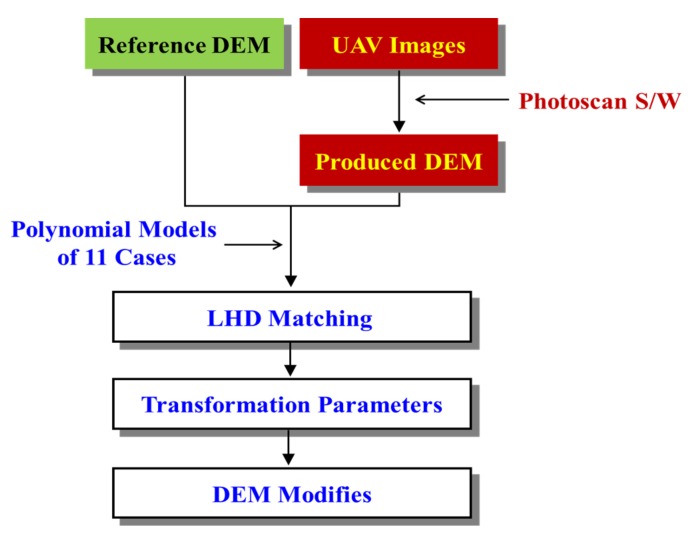
Flow-chart for proposed LHD matching and correction of DEM.

Figure 2 shows a flowchart of the steps used in the study. LHD matching is based on the three-dimensional (3-D) similarity equation (Equation (1)) that specifies the relationship between the reference and UAV DEMs (translations, rotation angles, and scale). The polynomial orders were selected after testing these functions.

A seven-parameter 3-D similarity transformation was used to express the geometric relationship between the reference DEM and UAV DEM points:

(1) $$\left[\begin{array}{c} X_{REF}\\ \begin{array}{c} Y_{REF}\\ Z_{REF} \end{array} \end{array}\right]=sR\left[\begin{array}{c} X_{UAV}\\ \begin{array}{c} Y_{UAV}\\ Z_{UAV} \end{array} \end{array}\right]+\left[\begin{array}{c} X_{T}\\ \begin{array}{c} Y_{T}\\ Z_{T} \end{array} \end{array}\right]$$

where $X_{REF}$, $Y_{REF}$, and $Z_{REF}$ are the 3-D coordinates of the reference DEM; and, $X_{UAV}$, $Y_{UAV}$, and $Z_{UAV}$ are the 3-D coordinates of the UAV DEM. $X_{REF}$, $Y_{REF}$ and $X_{UAV}$, $Y_{UAV}$ are the same-plane coordinates, whereas $Z_{REF}$ and $Z_{UAV}$ are the different heights.$\;X_{T}$, $Y_{T}$, and $Z_{T}$ are the translations, s is a scale factor, and R =$\;\left[\begin{array}{ccc} r_{11} & r_{12} & r_{13}\\ r_{21} & r_{22} & r_{23}\\ r_{31} & r_{32} & r_{33} \end{array}\right]$ is an orthogonal rotation matrix that contains the rotational angles (ω, φ, κ) with respect to the X, Y, and Z axes, respectively. $X_{T}=X_{0}+X_{1}\cdot l+X_{2}\cdot l^{2}+X_{3}\cdot l^{3}$, $Y_{T}=Y_{0}+Y_{1}\cdot l+Y_{2}\cdot l^{2}+Y_{3}\cdot l^{3}$, $Z_{T}=Z_{0}+Z_{1}\cdot l+Z_{2}\cdot l^{2}+Z_{3}\cdot l^{3}$, $\omega =\omega_{0}+\omega_{1}\cdot l+\omega_{2}\cdot l^{2}+\omega_{3}\cdot l^{3}$, $\varphi =\varphi_{0}+\varphi_{1}\cdot l+\varphi_{2}\cdot l^{2}+\varphi_{3}\cdot l^{3}$, $\kappa =\kappa_{0}+\kappa_{1}\cdot l+\kappa_{2}\cdot l^{2}+\kappa_{3}\cdot l^{3}$. l is the line as grid space of the DEM, the direction of which is the same as the flight path. $X_{0},X_{1},X_{2},X_{3}$, $Y_{0},Y_{1},Y_{2},Y_{3}$, $Z_{0},Z_{1},Z_{2},Z_{3}$, and $\omega_{0},\omega_{1},\omega_{2},\omega_{3}$, $\varphi_{0},\varphi_{1},\varphi_{2},\varphi_{3}$, $\kappa_{0},\kappa_{1},\kappa_{2},\kappa_{3}$ are the polynomial coefficients of the grid lines.

Equation (1) can be linearized by Taylor series expansion, as shown in Equation (2), based on the theory of Rosenholm and Torlegård (1988):

(2) $$X_{REF}+\Delta X=s^{o}\left(r_{11}^{o}X_{UAV}+r_{12}^{o}Y_{UAV}+r_{13}^{o}Z_{UAV}\right)+X_{T}^{o}-\Delta \kappa Y_{UAV}+\Delta \varphi Z_{UAV}+\Delta sX_{UAV}+\Delta X_{T}\;Y_{REF}+\Delta Y=s^{o}\left(r_{21}^{o}X_{UAV}+r_{22}^{o}Y_{UAV}+r_{23}^{o}Z_{UAV}\right)+Y_{T}^{o}+\Delta \kappa X_{UAV}-\Delta \omega Z_{UAV}+\Delta sY_{UAV}+\Delta Y_{T}\;Z_{REF}+\Delta Z=s^{o}\left(r_{31}^{o}X_{UAV}+r_{32}^{o}Y_{UAV}+r_{33}^{o}Z_{UAV}\right)+Z_{T}^{o}-\Delta \varphi X_{UAV}+\Delta \omega Y_{UAV}+\Delta sZ_{UAV}+\Delta Z_{T}$$

where the index *^o^* indicates an initial value. $\Delta X_{T}=\Delta X_{0}+\Delta X_{1}\cdot l+\Delta X_{2}\cdot l^{2}+\Delta X_{3}\cdot l^{3}$, $\Delta Y_{T}=\Delta Y_{0}+\Delta Y_{1}\cdot l+\Delta Y_{2}\cdot l^{2}+\Delta Y_{3}\cdot l^{3}$, $\Delta Z_{T}=\Delta Z_{0}+\Delta Z_{1}\cdot l+\Delta Z_{2}\cdot l^{2}+\Delta Z_{3}\cdot l^{3}$, $\Delta \omega =\Delta \omega_{0}+\Delta \omega_{1}\cdot l+\Delta \omega_{2}\cdot l^{2}+\Delta \omega_{3}\cdot l^{3}$, $\Delta \varphi =\Delta \varphi_{0}+\Delta \varphi_{1}\cdot l+\Delta \varphi_{2}\cdot l^{2}+\Delta \varphi_{3}\cdot l^{3}$, $\Delta \kappa =\Delta \kappa_{0}+\Delta \kappa_{1}\cdot l+\Delta \kappa_{2}\cdot l^{2}+\Delta \kappa_{3}\cdot l^{3}$. 

Equation (2) can be rewritten in terms of the ΔX and ΔY portions, as in Equation (3):

(3) $$\Delta X=\Delta X_{T}-\Delta \kappa Y_{UAV}+\Delta \varphi Z_{UAV}+\Delta sX_{UAV}$$

$$\Delta Y=\Delta Y_{T}+\Delta \kappa X_{UAV}-\Delta \omega Z_{UAV}+\Delta sY_{UAV}$$

If the *Z*-axis of the reference DEM is the same as that of the UAV DEM, then $Z_{REF}=f\left(X_{UAV},Y_{UAV}\right)$. The equation can be linearized as in Equation (4).

(4) $$\Delta Z=\frac{\partial f}{\partial X_{UAV}}\Delta X+\frac{\partial f}{\partial Y_{UAV}}\Delta Y$$

ΔX  and ΔY from Equation (3) are substituted in Equation (4), which is then expressed, as follows:

(5) $$\Delta Z=\frac{\partial f}{\partial X_{UAV}}\left(\Delta X_{T}-\Delta \kappa Y_{UAV}+\Delta \varphi Z_{UAV}+\Delta sX_{UAV}\right)+\frac{\partial f}{\partial Y_{UAV}}\left(\Delta Y_{T}+\Delta \kappa X_{UAV}-\Delta \omega Z_{UAV}+\Delta sY_{UAV}\right)$$

ΔZ of Equation (5) is substituted into the Z term in Equation (2), and the transformation parameters are finally determined by Equation (6):

(6) $$Z_{REF_{i}}-Z_{REF_{i}}^{o}=-\frac{\partial f_{i}}{\partial X_{UAV_{i}}}\Delta X_{T}-\frac{\partial f_{i}}{\partial Y_{UAV_{i}}}\;\Delta Y_{T}+\Delta Z_{T}$$

$$+\left[-\frac{\partial f_{i}}{\partial X_{UAV_{i}}}X_{UAV_{i}}-\frac{\partial f_{i}}{\partial Y_{UAV_{i}}}\;Y_{UAV_{i}}+Z_{UAV_{i}}\right]\Delta s$$

$$+\left(\;Y_{UAV_{i}}+\frac{\partial f_{i}}{\partial Y_{UAV_{i}}}\;Z_{UAV_{i}}\right)\Delta \omega$$

$$+\left(-X_{UAV_{i}}-\frac{\partial f_{i}}{\partial X_{UAV_{i}}}\;Z_{UAV_{i}}\right)\Delta \varphi$$

$$+\left(\;\frac{\partial f_{i}}{\partial X_{UAV_{i}}}Y_{UAV_{i}}-\frac{\partial f_{i}}{\partial Y_{UAV_{i}}}\;X_{UAV_{i}}\right)\Delta \kappa$$

where *i* = 1,2, …, n (n = number of points), and $Z_{REF_{i}}^{o}=s^{o}\left(r_{31}^{o}X_{UAV_{i}}+r_{32}^{o}\;Y_{UAV_{i}}+r_{33}^{o}\;Z_{UAV_{i}}\right)+Z_{T}^{o}$, which is the initial height calculated while using the initial polynomial parameters. In the Z term in Equation (1), $\frac{\partial f_{i}}{\partial X_{UAV_{i}}\;}\;$and $\frac{\partial f_{i}}{\partial Y_{UAV_{i}}}\;$are the slopes with respect to the X and Y directions of the two grid spaces in the produced DEM. If the value of the slope is close to zero, the accuracy of the parameters is low, as shown in Equation (6). 

Δ is computed by the least squares method and iteration ($Z_{T}^{1}=Z_{T}^{o}+\Delta Z_{T}$; $\Delta Z_{T}=\Delta Z_{0}+\Delta Z_{1}\cdot l+\Delta Z_{2}\cdot l^{2}+\Delta Z_{3}\cdot l^{3}$) to calculate the transformation parameters. The transformation parameters are then determined until the Δ terms are nearly zero. LHD matching uses the height difference at the same-plane locations between the two DEMs. It is important that the height difference ($d_{i}$) of any point (i) should not be largely different from those of other points. Therefore, we first determined the same-plane positions between the two DEMs within a grid interval and then calculated the height differences at those points before applying the LHD matching. Only the point where $d_{i}$ is within 2 *σ* (*σ* is the standard deviation) of $d_{m}\;$can participate in the LHD matching operation to limit the gross error point:

(7) $$d_{m}=\frac{d_{1}+\cdots +d_{n}}{n}$$

where $d_{m}\;$is the average height difference between all of the points in the search.

## 3. Experiment and Results

**Figure 3:**
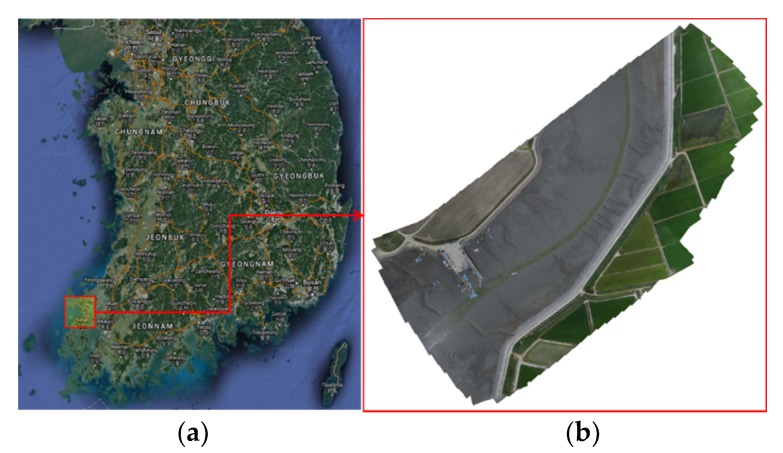
Test location (**a**) and orthomosaic image taken by the camera onboard the UAV (**b**).

**Figure 4:**
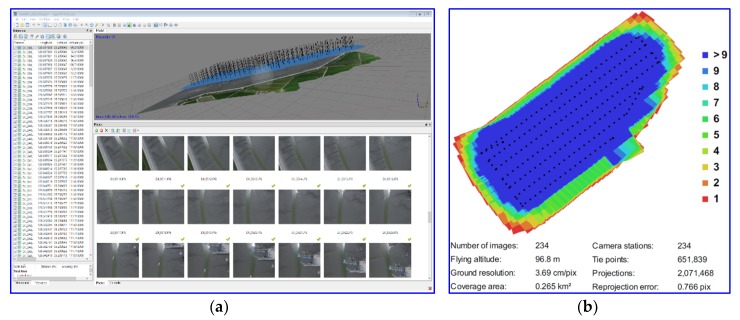
PhotoScan process for DEM (**a**); camera locations and image overlap in PhotoScan processing report (**b**).

**Figure 5:**
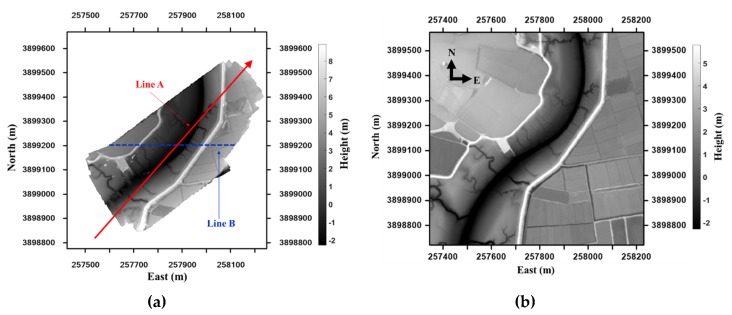
UAV DEM (**a**), Line A is the direction for the polynomial models of the transformation parameters, Line B is the profile for error check, and light detection and ranging (LiDAR) DEM (**b**).

The test site chosen to apply the above-mentioned method was a tidal flat area in Hampyung Bay, which is located on the west coast of the Korean Peninsula (Figure 3). The site is an embayment situated in a deep inland from the west coast and it is a unique geographic feature that is formed without large rivers. The UAV images were acquired while using a DJI Phantom 4, as shown in Table 1, at 2:20 pm on August 12, 2016, at the onset of high tide from low tide. The photogrammetric process was implemented by the PhotoScan to obtain the DEM of the site. The camera was self-calibrated during the alignment of the image without GCPs. At this stage, corresponding points were identified on the images and matched; in addition, the position of the camera for each image from onboard global navigation satellite system data and camera calibration parameters were automatically refined and stored in the EXIF metadata of the images [25]. With the dense stereo matching of the PhotoScan, a 10 cm resolution DEM was generated by the UAV images (Figure 4). Figure 5 shows the produced UAV DEM and LiDAR DEM (resolution 1 m; obtained in 2011) used as the reference DEM. The UAV and LiDAR DEMs are both digital surface models that represent the mean sea level elevations of the reflective surfaces of all features. The difference between the two DEMs due to vegetation is not high, because most of the test area is covered by tidal flats and the data includes roads, paddy fields, and wild land.

The IOPs and EOPs of the camera influence the accuracy of the DEM derived from UAV photogrammetry [26]. The EOPs were calculated by the position of the GPS equipped on the DJI Phantom 4 and bundle adjustment of the PhotoScan; however, these were not accurate. GCPs must be installed and surveyed on the tidal flat area to obtain the accurate DEM by correction of the IOPs and EOPs. However, the surveying of GCPs is almost impossible, as access to the muddy tidal flat of the Southwest coast is very difficult. Therefore, in this study, the produced DEM was corrected while using a pre-secured LiDAR DEM with a new LHD DEM matching method. In the experiment, we applied the diagonal direction (Line A in Figure 5) as the flight path of the UAV DEM to determine the polynomial models of the transformation. Next, we used the new LHD matching method with the following eleven cases to obtain the transformation parameters:

Case 1: 7 parameters ($X_{0},\;Y_{0},Z_{0},\;\;\omega_{0},\;\varphi_{0},\;\kappa_{0},s$)
Case 2: 10 parameters ($X_{0},\;Y_{0},Z_{0}$, $X_{1},\;Y_{1},Z_{1},$ $\omega_{0}$,$\varphi_{0},\kappa_{0},s$)
Case 3: 13 parameters ($X_{0},\;Y_{0},Z_{0}$, $X_{1},\;Y_{1},Z_{1},\omega_{0}$,$\varphi_{0},\kappa_{0},\omega_{1}$,$\varphi_{1},\kappa_{1},s$)
Case 4: 13 parameters ($X_{0},\;Y_{0},Z_{0}$, $X_{1},\;Y_{1},Z_{1},X_{2},\;Y_{2},Z_{2},$ $\omega_{0}$,$\varphi_{0},\kappa_{0},s$)
Case 5: 16 parameters ($X_{0},\;Y_{0},Z_{0}$, $X_{1},\;Y_{1},Z_{1},X_{2},\;Y_{2},Z_{2},$ $\omega_{0}$,$\varphi_{0},\kappa_{0},\;\;\omega_{1}$,$\varphi_{1},\kappa_{1},s$)
Case 6: 19 parameters ($X_{0},\;Y_{0},Z_{0}$, $X_{1},\;Y_{1},Z_{1},X_{2},\;Y_{2},Z_{2},$ $\omega_{0}$,$\varphi_{0},\kappa_{0},\omega_{1}$,$\varphi_{1},\kappa_{1},\omega_{2}$,$\varphi_{2},\kappa_{2},s$)
Case 7: 22 parameters ($X_{0},\;Y_{0},Z_{0}$,$X_{1},\;Y_{1},Z_{1},\;X_{2},\;Y_{2},Z_{2},\;\omega_{0}$,$\varphi_{0},\kappa_{0},\;\omega_{1}$,$\varphi_{1},\kappa_{1},\omega_{2}$,$\varphi_{2},\kappa_{2},\omega_{3}$, $\varphi_{3},\kappa_{3},s$)
Case 8: 16 parameters ($X_{0},\;Y_{0},Z_{0}$, $X_{1},\;Y_{1},Z_{1},X_{2},\;Y_{2},Z_{2},X_{3},Y_{3},Z_{3}$,$\;\omega_{0}$,$\varphi_{0},\;\kappa_{0},s$)
Case 9: 19 parameters ($X_{0},\;Y_{0},Z_{0}$, $X_{1},\;Y_{1},Z_{1},X_{2},\;Y_{2},Z_{2},X_{3},Y_{3},Z_{3}$, $\omega_{0}$,$\varphi_{0},\kappa_{0},\;\omega_{1}$,$\varphi_{1},\;\kappa_{1},s$)
Case 10: 22 parameters ($X_{0},\;Y_{0},Z_{0}$,$\;X_{1},\;Y_{1},Z_{1},X_{2},\;Y_{2},Z_{2},X_{3},Y_{3},Z_{3}$,$\;\omega_{0}$,$\varphi_{0},\kappa_{0},\omega_{1}$,$\varphi_{1},\;\kappa_{1},\omega_{2}$,$\varphi_{2},\kappa_{2},s$)
Case 11: 25 parameters ($X_{0},\;Y_{0},Z_{0}$,$\;X_{1},\;Y_{1},Z_{1},X_{2},\;Y_{2},Z_{2},\;X_{3},Y_{3},Z_{3}$, $\omega_{0}$,$\varphi_{0},\kappa_{0},\omega_{1}$,$\varphi_{1},\kappa_{1},\omega_{2}$, $\varphi_{2},\kappa_{2},\omega_{3}$,$\varphi_{3},\kappa_{3},s$)

Table 2 shows the estimated constant terms (six parameters) of the polynomial model in all cases. The results of scale factor are almost 1.0 in all cases; therefore, we have omitted the scale representation. Standard deviations of shift and rotation parameters that are determined by the least squares solution do not exceed 0.3 m and 0.1°, respectively, except Cases 10 and 11. In particular, the six parameters of Case 4 and Case 8 are similar; however, Case 4 has a higher accuracy than Case 8. Overall, Cases 10 and 11 are the least reliable. The degree of the polynomial model does not necessarily have to be high or overparameterized for using it with this method; this shows that the reliability of the parameter can be decreased.

**Figure 6:**
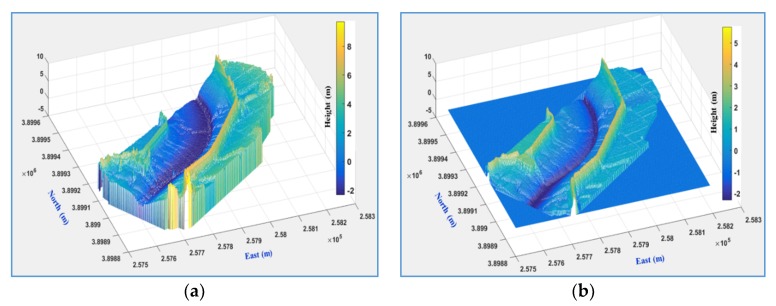
(**a**) UAV DEM and (**b**) LiDAR DEM.

The produced DEM (Figure 6a) was modified by the transformation parameters through the proposed LHD matching that was based on the eleven cases using LiDAR DEM as the reference DEM (Figure 6b). Table 3 shows a quantitative comparison of each modified DEM with LiDAR DEM. The error before DEM correction is 1.4 m with a maximum error of 7.8 m. After DEM correction, the RMSE and maximum error fell to 1.2 m and 6.8 m, respectively, for Case 1. The accuracy slightly improved from Case 1 to Case 3; the accuracy was not improved for Case 4. The error in the DEM is relatively large when compared to DEM resolution (10 cm) due to the inflow water from the tidal flat. There is a possibility that tie-point, self-calibration, and block-adjustment errors in PhotoScan due to the reflection of the underwater area during the process of stereo matching affect the bowl effect.

**Figure 7:**
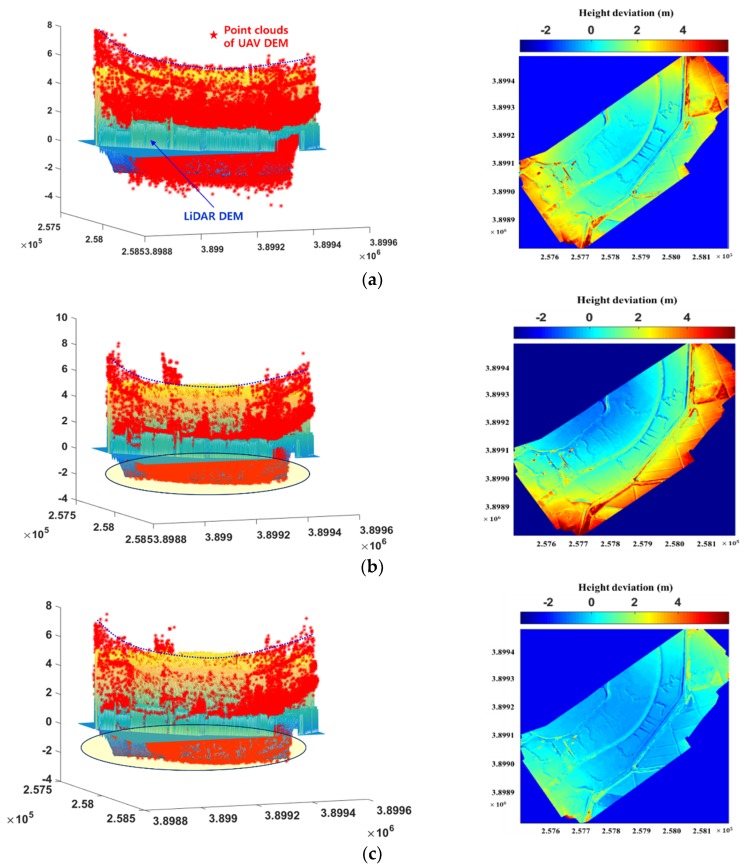

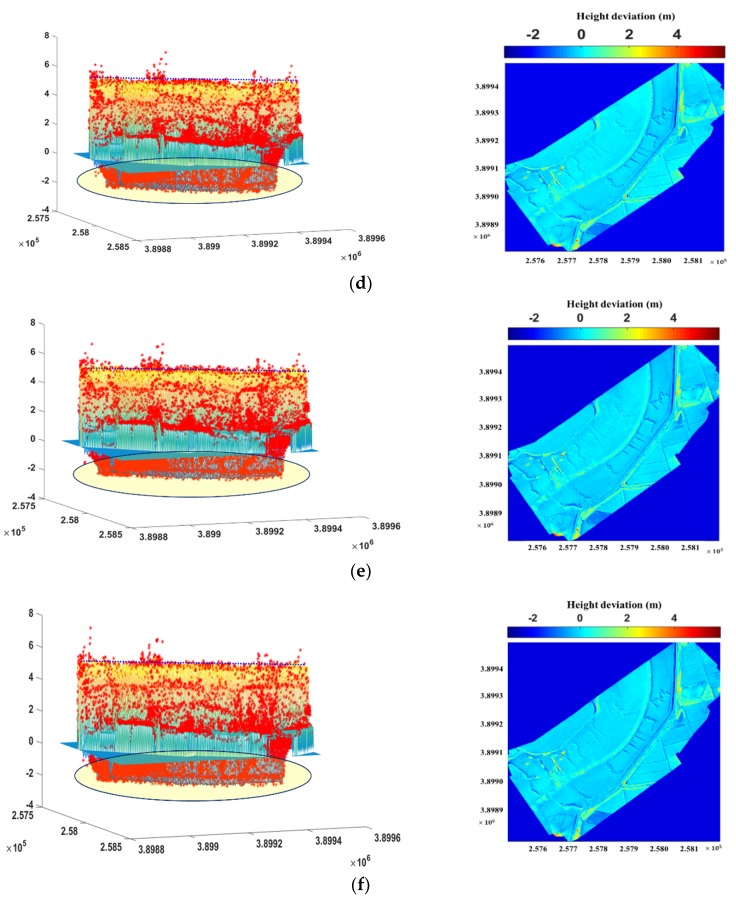
DEM (**a**) before correction and after correction for (**b**) Case 1, (**c**) Case 2, (**d**) Case 3, (**e**) Case 10, and (**f**) Case 11. The figures on the left are overlaid with UAV point clouds and LiDAR mesh; the figures on the right are color maps of height deviations between UAV DEMs and LiDAR DEM (in meters).

Figure 7 shows a visual comparison before and after correction for Cases 1, 2, 3, 10, and 11. The left side of Figure 7 shows the point clouds from the UAV images on the mesh of the LiDAR DEM in the lateral view of the 3-D surface, and the right side shows a comparison of the deviations in height between the UAV DEMs and LiDAR DEM. 

On the left side of Figure 7, it can be seen that the original UAV DEMs (Figure 7a), and the corrected Cases 1 and 2 DEMs (Figure 7b,c), are almost parabolic in shape (dashed lines), and point clouds are inconsistent with the LiDAR mesh (elliptical shape), whereas the corrected DEMs in Cases 3, 10, and 11 (Figure 7d–f) closely match the LiDAR mesh. In the color maps on the right side of Figure 7, Cases 1 and 2 DEMs are lower than the LiDAR DEM in the central part and higher on the outer part, whereas the heights of the DEMS for Cases 3, 10, and 11 are closer to that of the LiDAR DEM. As the corrected DEMs for Cases 3–11 were almost similar, these comparisons were omitted.

**Figure 8:**
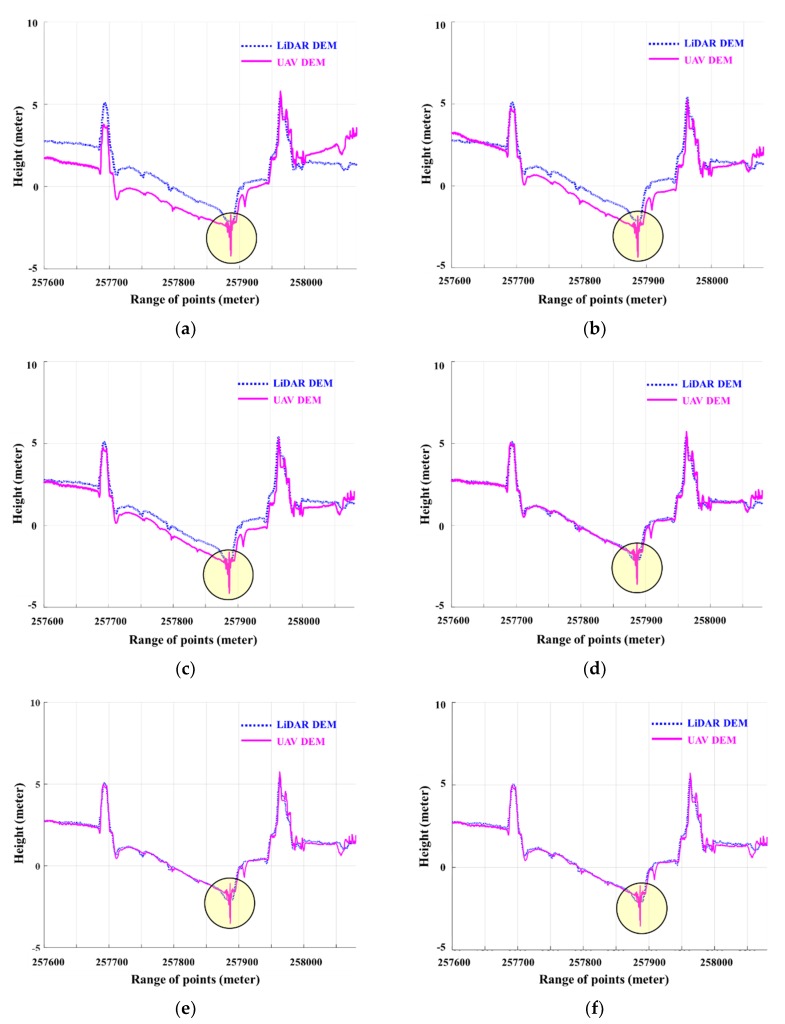
(**a**) DEM profiles before and after correction using the UAV images and LiDAR DEM for (**b**) Case 1, (**c**) Case 2, (**d**) Case 3, (**e**) Case 10, and (**f**) Case 11. The circled section shows the gross error in the UAV DEM due to tide inflows.

For a detailed comparison of the corrected DEMs, Figure 8 presents the height deviation in each of the cases between the LiDAR DEM and UAV DEM before and after the correction in the Line B profile, as shown in Figure 5. The analysis of accuracy was performed while using the height deviation between the two profiles of the corrected DEMs and LiDAR DEM.

In Figure 8, the errors in Cases 1 and 2 were reduced when compared to the original UAV DEM. However, the error due to the bowl effect still existed even after the correction by applying parameters 7 and 10. Yet, it was observed that this error was removed by applying parameter 13 to Case 3. Therefore, if the transformation parameter is applied by the LHD method, which does not take the polynomial as a flight path of the DEM without the GCP, the bowl effect cannot be eliminated. The optimal parameters were selected from among the 11 cases by calculating the RMSE in the height deviation between the two DEMs after correction (Table 3 and Table 4). In particular, the error sharply decreases from Case 3 to approach the spatial resolution of the UAV image (10 cm) as compared to that before the correction, and the difference in accuracy from Case 3 to Case 11 is less than cm, as shown in Table 4. Applying more parameters showed no significant effect. Table 2 shows that the parameters tended to become slightly inferior as higher-order polynomials were used (Case 10 and Case 11). Therefore, in this study, it was surmised that the optimal parameter to correct the UAV-made DEM while using the reference DEM and proposed LHD matching method was Case 4, which was similar to the bowl effect geometry. It is the secondary curved polynomial with constant rotation having the principal axis in the direction of line, as shown in Figure 5. The experimental results verified that the proposed technique reduced the error of the DEM that was produced by the UAV using only the reference DEM and also eliminated the error due to the bowl effect without requiring GCPs.

## 4. Conclusions

It is important to obtain a DEM of a tidal flat periodically in order to monitor its environmental condition. It is difficult to acquire GCPs in the tidal flat, although UAV images for accurate and economic DEM production are appropriate. Therefore, we proposed a polynomial LHD DEM matching method by which the position and rotation distortion and bowl effect error were removed, without the requirement of GCPs, in a tidal flat DEM produced from UAV images using a photogrammetry software. The test site chosen to apply the proposed method was a tidal flat area in Hampyung Bay that was located on the west coast of the Korean Peninsula. The experiments were conducted in 11 cases with different correction models to determine the optimal LHD polynomial model. In the experimental investigations, we proposed an optimal model, in which the translation parameters were second-order polynomials and rotation parameters were constant as the number of DEM grids increased in the flight direction. The RMSE in the height of this case was nearly 15 cm. This result was close to the DEM resolution (10 cm) from the acquired UAV image. The experimental results showed that the proposed method could reduce the error in the tidal flat DEM while using only the reference DEM and eliminated the bowl effect error. This method is very effective for correction in the DEM produced by UAV images obtained in inaccessible areas where GCP survey is difficult.

The limitation of the method that is proposed in this paper is that the accuracy of the corrected DEM is affected by the accuracy of the reference DEM. As the height accuracy of LiDAR DEM used as the reference DEM is 15–20 cm, the UAV DEM can be regarded as having similar or lower accuracy, and it is necessary to use an independent reference point to evaluate the actual accuracy of the corrected UAV DEM.

## Figures and Tables

**Table 1:** Technical data of Phantom 4 and camera.

Items		Parameters
Phantom 4	Weight	1380 g
	Max. speed	20 m/s
	Flight time	28 min
	GNSS	GPS, GLONASS
Camera	Focal length	3.61 mm
	FOV	94°
	Sensor size	12.4 M (4000 × 3000)

**Table 2:** Six transformation parameters and their standard deviations.

Case #	Parameters					
	$X_{0}/\sigma_{X_{0}}\left(m\right)$	$Y_{0}/\sigma_{Y_{0}}(m)$	$Z_{0}/\sigma_{Z_{0}}(m)$	$\omega_{0}/\sigma_{\omega_{0}}\;({}^{\circ})$	$\varphi_{0}/\sigma_{\varphi_{0}}({}^{\circ})$	$\kappa_{0}/\sigma_{\kappa_{0}}({}^{\circ})$
1	−0.59/0.012	0.33/0.013	−0.03/0.004	0.34/0.002	0.32/0.002	−0.02/0.003
2	−0.18/0.070	0.66/0.074	10.85/0.131	1.19/0.008	−0.51/0.008	−0.02/0.015
3	−0.59/0.012	0.33/0.013	11.27/0.004	0.34/0.002	0.32/0.001	−0.02/0.003
4	**0.42/0.057**	**1.78/0.061**	**8.88/0.074**	**1.34/0.011**	−**0.64/0.012**	**0.01/0.008**
5	1.86/0.085	0.19/0.080	2.97/0.225	0.81/0.018	−0.18/0.017	−0.21/0.060
6	2.22/0.087	0.25/0.080	2.94/0.227	0.84/0.019	−0.15/0.017	−0.46/0.060
7	1.95/0.098	0.41/0.085	3.65/0.233	1.21/0.021	−0.05/0.019	−0.29/0.061
8	−**2.27/0.115**	**2.67/0.120**	**8.87/0.076**	**1.35/0.042**	−**0.64/0.046**	**0.03/0.012**
9	−0.86/0.130	1.16/0.127	2.93/0.226	0.81/0.044	−0.17/0.043	−0.20/0.060
10	−0.48/0.233	1.63/0.198	15.76/0.600	1.85/0.083	−1.18/0.072	−0.19/0.241
11	−1.07/0.237	1.86/0.198	17.79/0.610	2.32/0.083	−1.18/0.071	0.05/0.243

**Table 3:** Height deviations between the LiDAR DEM and UAV DEM before and after correction.

Correction	Before	After: Case #										
		1	2	3	4	5	6	7	8	9	10	11
RMSE (m)	1.37	1.24	1.21	1.12	1.11	1.11	1.10	1.11	1.10	1.10	1.10	1.10
Max (m)	7.84	6.76	6.28	5.99	6.00	5.98	5.96	5.95	5.98	5.95	5.95	5.94

**Table 4:** Height deviations between the two DEMs before and after correction on the line B profile in Figure 5.

Correction	Before	After: Case #										
		1	2	3	4	5	6	7	8	9	10	11
RMSE (m)	0.956	0.517	0.475	0.150	0.149	0.153	0.156	0.166	0.152	0.157	0.149	0.163
Max (m)	2.258	2.218	2.060	1.468	1.440	1.385	1.393	1.426	1.457	1.403	1.414	1.451
